# The Impact of Severe COVID-19 on Plasma Antioxidants

**DOI:** 10.3390/molecules27165323

**Published:** 2022-08-21

**Authors:** Neven Žarković, Anna Jastrząb, Iwona Jarocka-Karpowicz, Biserka Orehovec, Bruno Baršić, Marko Tarle, Marta Kmet, Ivica Lukšić, Wojciech Łuczaj, Elżbieta Skrzydlewska

**Affiliations:** 1 Laboratory for Oxidative Stress (LabOS), Ruđer Bošković Institute, HR-10000 Zagreb, Croatia; 2 Department of Analytical Chemistry, Medical University of Bialystok, 15-089 Bialystok, Poland; 3 Clinical Department of Laboratory Diagnostics, Clinical Hospital Dubrava, HR-10000 Zagreb, Croatia; 4 Department of Internal Medicine, Clinical Hospital Dubrava, HR-10000 Zagreb, Croatia; 5 Department of Maxillofacial Surgery, Clinical Hospital Dubrava, HR-10000 Zagreb, Croatia; 6 School of Medicine, University of Zagreb, HR-10000 Zagreb, Croatia

**Keywords:** COVID-19, SARS-CoV-2, biomarkers, plasma, antioxidants, GSH system, Trx system, lipid peroxidation, 4-HNE-protein adducts

## Abstract

Several studies suggested the association of COVID-19 with systemic oxidative stress, in particular with lipid peroxidation and vascular stress. Therefore, this study aimed to evaluate the antioxidant signaling in the plasma of eighty-eight patients upon admission to the Clinical Hospital Dubrava in Zagreb, of which twenty-two died within a week, while the other recovered. The differences between the deceased and the survivors were found, especially in the reduction of superoxide dismutases (SOD-1 and SOD-2) activity, which was accompanied by the alteration in glutathione-dependent system and the intensification of the thioredoxin-dependent system. Reduced levels of non-enzymatic antioxidants, especially tocopherol, were also observed, which correlated with enhanced lipid peroxidation (determined by 4-hydroxynonenal (4-HNE) and neuroprostane levels) and oxidative modifications of proteins assessed as 4-HNE-protein adducts and carbonyl groups. These findings confirm the onset of systemic oxidative stress in patients with severe SARS-CoV-2, especially those who died from COVID-19, as manifested by strongly reduced tocopherol level and SOD activity associated with lipid peroxidation. Therefore, we propose that preventive and/or supplementary use of antioxidants, especially of lipophilic nature, could be beneficial for the treatment of COVID-19 patients.

## 1. Introduction

One of the basic conditions of the physiological state of the body is the redox balance at the level of cells and tissues, including body fluids [1,2]. This is due to the fact that the generation of reactive oxygen species (ROS) remains at a relatively low level, and their action is balanced by the action of a complex of enzymatic and non-enzymatic antioxidants involved in the maintenance of redox homeostasis [3,4]. However, in response to the invasion of pathogens, the host organism activates leukocytes, which, as a result of an increased activity of pro-oxidative enzymes, generate large amounts of ROS [5,6]. ROS modify both the host organism’s resistance and susceptibility to infections [7] since, due to their non-specific nature and unique reactivity in larger amounts, ROS become harmful to both the pathogen and the host [8]. Overproduction of ROS promotes the degradation of pathogens, and thus protects the body against their effects; however, it is known that their elevated level may enhance cytotoxicity and damage the host’s molecules/cells/organs, while their reduced level may favor the survival and spread of pathogens [7,9]. The consequences are similar in both situations, but reduced ROS levels usually lead to increased host mortality [9]. The generated ROS modulate the signal transduction cascade and enhance the immune functions of lymphocytes [10]. Moreover, infections are usually accompanied by inflammation and a decrease in the effectiveness of endogenous antioxidant defense mechanisms, which ultimately promotes the development of oxidative stress observed in viral diseases [11,12]. On the other hand, under oxidative stress conditions, oxidative modifications of the basic components of cells/biological fluids, such as DNA, lipids, and proteins occur, which may alter their functionality, including the promotion of inflammation, which is a significant consequence of the host’s immune response [13,14]. Consequently, the disruption of host redox homeostasis by pathogens leads to a modification of both cellular metabolism and intra- and extracellular signaling.

Recent studies indicate that infections with viruses from the Coronaviridae family also contribute to redox imbalance and the formation of oxidative stress in the host organism [15,16]. Immunohistochemical studies have shown that COVID-19 increases the level of the primary antioxidant enzyme, i.e., manganese superoxide dismutase (SOD-2), in cells of vital organs, including the kidneys [17]. However, the level of this enzyme was lower in the lungs and has only been detected in some inflammatory cells [17]. In addition, it was suggested that the observed enhancement of total antioxidant capacity (TAC) in pregnant women with COVID-19 may be the body’s response to damage to cellular components caused by excess ROS [18]. One of the main consequences of oxidative stress is the modification of physiological lipid metabolism. This is a direct consequence of an increased level of ROS as well as activity of lipid-metabolizing enzymes resulting from oxidative stress [19]. As a result of ROS-dependent lipid peroxidation, the level of PUFAs oxidative fragmentation products enhances with the formation of low-molecular, reactive aldehydes, including 4-hydroxynonenal (4-HNE), which, as an electrophilic compound, easily reacts with nucleophilic fragments of proteins to form 4-HNE-protein adducts [20,21]. A clear dynamic variation in the level of these adducts was observed in the blood of COVID-19 survivors [22]. At the same time, immunohistochemical studies confirmed the high level of HNE-protein adducts in internal organs, apart from the kidneys of deceased patients [17]. However, in the case of the bodies of those patients who did not survive the infection, the level of these adducts did not change during the course of the disease, while a positive correlation was found between the level of these adducts and the level of autoantibodies against oxidized LDL, which indicates a malfunctioning in the patient’s immune system. Therefore, it has been suggested that the elevated HNE level was most likely caused by vascular stress (similar to sepsis) rather than the inflammation accompanying the infection [22].

In addition, higher death rates from SARS-CoV-2 (COVID-19) coronavirus infection have been found among the elderly and people with comorbidities, suggesting that these factors make these people more sensitive to infectious agents, such as the SARS-CoV-2 coronavirus. It is believed that particularly disturbed redox homeostasis and the resulting oxidative stress may be responsible for increased individual susceptibility to the pathogen. Moreover, there are studies showing that some proteins, such as type 3-cystatins, responsible for inhibiting viral peptidases are more abundant in survivors, while inter-α-trypsin inhibitors involved in the inflammatory process are found in varying amounts in individuals, survivors, and those who did not survive COVID-19 [23].

Therefore, taking into account the above-mentioned data, especially regarding the enhanced generation of ROS accompanying viral infections, it seems important to determine the direction of changes in the host metabolic parameters in the course of COVID-19 infection, and in particular the metabolic differentiation between surviving and deceased patients. Moreover, it is important to identify the metabolic consequences resulting from oxidative stress, especially on lipids, leading to the death of patients. Therefore, the aim of this study was to obtain comprehensive data on antioxidant signaling under redox stress in the blood plasma of patients with COVID-19.

## 2. Results

The studies of redox balance parameters and oxidative stress parameters were carried out with the use of plasma collected from patients who survived COVID-19 (66) and those who died as a result of COVID-19 (22), as well as healthy people (33), whose demographic data are presented in Table 1.

The data obtained from the analysis of plasma from healthy subjects and hospitalized patients with COVID-19 show that the course of infection causes significant changes in level/activity of parameters related to the antioxidant action, as well as the level of lipid peroxidation products and protein modifications.

**Figure 1:**
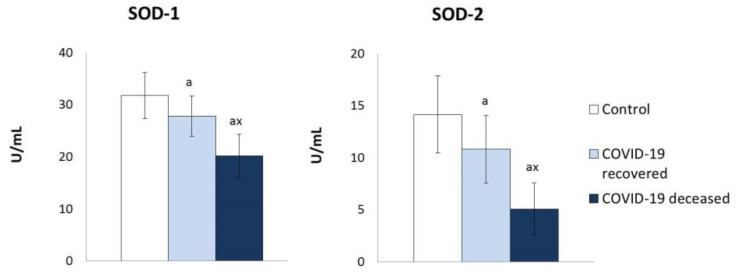
The influence of COVID-19 on the activity of superoxide dismutase-1 and superoxide dismutase-2 in the plasma of survivors and patients who died as a result of infection. Mean values ± SD are presented with statistically significant differences: a—vs. control group, *p* < 0.05; x—between patient groups, *p* < 0.05.

It was found that the previously demonstrated enhancement in ROS generation in plasma of COVID-19 patients is accompanied by a decrease in the activity of the basic enzymes responsible for the dismutation of the superoxide anion radicals; (Cu,Zn-SOD—SOD-1 and Mn-SOD—SOD-2), including patients who survived and considerably stronger patients who have died (Figure 1).

**Figure 2:**
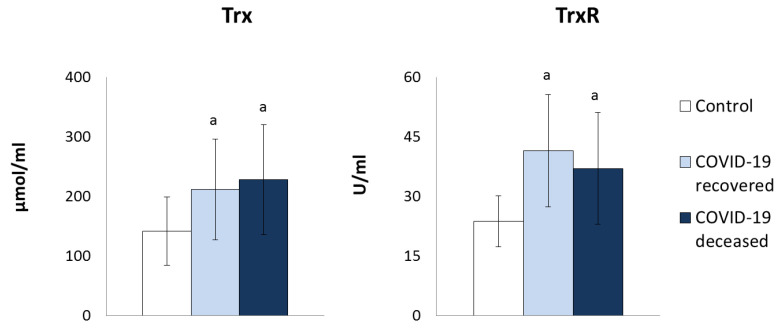
The influence of COVID-19 on the thioredoxin-dependent antioxidant system in the plasma of survivors and patients who died as a result of infection. Mean values ± SD are presented with statistically significant differences: a—vs. control group, *p* < 0.05.

**Figure 3:**
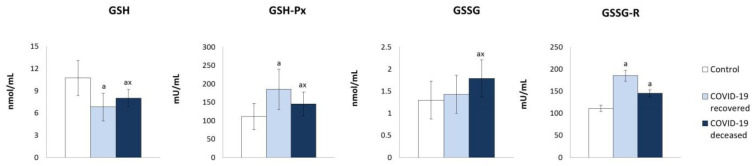
The influence of COVID-19 on the glutathione-dependent antioxidant system in the plasma of survivors and patients who died as a result of infection. Mean values ± SD are presented with statistically significant differences: a—vs. control group, *p* < 0.05; x—between patient groups, *p* < 0.05.

**Figure 4:**
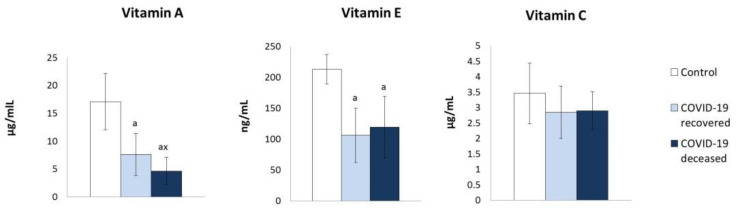
The influence of COVID-19 on the vitamins A, E, and C level in the plasma of survivors and patients who died as a result of infection. Mean values ± SD are presented with statistically significant differences: a—vs. control group, *p* < 0.05; x—between patient groups, *p* < 0.05.

On the other hand, the parameters of the antioxidant systems dependent on thioredoxin (Trx) and glutathione (GSH) underwent significant quantitative changes (Figure 2 and Figure 3). Both thioredoxin (Trx) protein level and thioredoxin reductase (TrxR) activity of the thioredoxin system (Trx and TrxR) were significantly elevated in the plasma of COVID-19 patients compared to healthy subjects. However, the level of reduced glutathione (GSH) was reduced, while the level of oxidized glutathione (GSSG) was elevated, particularly in plasma of surviving patients and with tendency to increase in patients who have died (Figure 3). However, the activity of both enzymes glutathione peroxidase (GSHPx) and glutathione reductase (GSSG-R) regulating GSH/GSSG levels was enhanced in patients, but tended to reduce GSSG-R activity in the plasma of patients who died compared to survivors. Moreover, the decrease in the level of GSH in the plasma of patients was accompanied by a reduction in the level of lipophilic antioxidants, such as vitamins A and E (Figure 4).

On the one hand, the above data suggest the modulating effect of COVID-19 on proteins with antioxidant activity (which, in turn, may indicate the importance of Trx in the course of COVID-19 related to gene regulation and protein expression mediated by the nuclear factor kappa-Β (NFκB) transcription factor). On the other hand, a reduction in antioxidant efficiency is observed due to the reduction of glutathione (GSH) levels and antioxidant vitamins.

**Figure 5:**
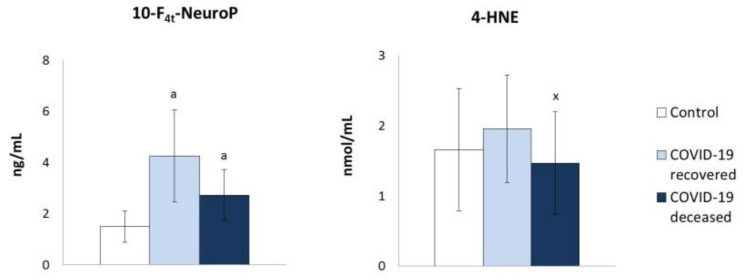
The effect of COVID-19 on the level of lipid peroxidation products estimated as lipid oxidative cyclisation product 10-F4t-NeuroP and lipid oxidative fragmentation product (4-HNE) in the plasma of survivors and patients who died as a result of infection. Mean values ± SD are presented with statistically significant differences: a—vs. control group, *p* < 0.05; x—between patient groups, *p* < 0.05.

**Figure 6:**
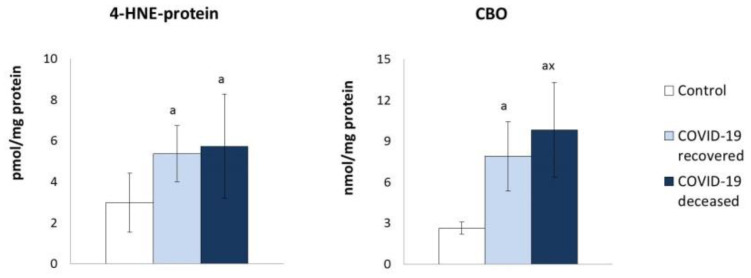
The effect of COVID-19 on the level of 4-HNE protein adducts and protein carbonyl groups (CBO) in the plasma of survivors and patients who died as a result of infection. Mean values ± SD are presented with statistically significant differences: a—vs. control group, *p* < 0.05; x—between patient groups, *p* < 0.05.

Taking into account the literature data on the enhanced ROS generation in the blood of COVID-19 patients [24] and the observed altered antioxidant capacity in the blood of patients in this study, it would be difficult to quantify the redox balance changes and the metabolic effects of these changes. Therefore, the level of lipid peroxidation and protein modification products was assessed (Figure 5). This allowed for the conclusion that the plasma of COVID-19 patients was characterized by increased lipid peroxidation assessed on the basis of the level of 4-hydroxynonenal (4-HNE) and neuroprostanes (10-F4t-NeuroP), while the level of 10-F4t-NeuroP in the surviving patients was almost 3 times higher than in the control group. However, their level diminished in the plasma of patients who did not survive the infection. At the same time, a higher tendency to form 4-HNE adducts with proteins was found (Figure 6), which may explain the lower increase in the level of 4-HNE than in the case of neuroprostanes. An elevated level of carbonyl groups was also observed in plasma proteins, which is an indicator of the oxidative stress and its influence on the protein structure.

Variable importance of the factors in the projection (VIP) coefficient revealed that six parameters analyzed in this study lead to the separation of examined groups (control, COVID-19 recovered patients, and COVID-19 deceased patients). These parameters included vitamin E, GSSG-R, 10-F4t-NeuroP, GSH, vitamin A, GSH-Px, and adducts of 4-HNE-proteins (Supplementary Materials, Figure S3).

## 3. Discussion

During infection, the host cells produce, receive, and react to signaling molecules whose level enhances in response to the presence of the pathogen in the body [25,26]. Consequently, as a result of the increased activity of pro-oxidative enzymes, the host organism activates leukocytes, which results in the production of a large amount of reactive oxygen and nitrogen species (ROS and RNS), which play a key role in the host’s immune defense against pathogens [5,6]. Literature data show that patients with COVID-19 overexpress NADPH-2 oxidase (NOX-2), which is responsible for the production of superoxide anion and the intensification of oxidative stress [27]. However, it is known that inhibiting the activity of this oxidase in macrophages improves phenotypes of diseases [28]. Since oxidative stress is usually accompanied by inflammation, which is a consequence of the interplay of transcription factors, such as nuclear factor erythroid-derived 2-like 2 (Nrf2) and nuclear factor kappa B (NFkB), this leads to enhanced generation of pro-inflammatory cytokines, such as interlukin-6 (IL-6). Inflammation in COVID-19 patients was additionally confirmed by the elevated C-reactive protein (CRP) value, which was significantly higher in patients who died than in those who recovered. In the case of COVID-19, due to the rising viral load, there is a rapid increase in inflammatory monocytes and neutrophils in the lungs, activating cytokines and chemokines that continuously and irreversibly affect lung tissue, thereby enhancing risk of death [29]. On the other hand, activation of neutrophils can also be caused by excessive activation of platelets, which can also lead to thrombo-inflammation [30]. Platelet levels were especially higher in patients who recovered, while those who died had more platelets than healthy individuals, but less than those who recovered. This may be related to the fact that the levels of circulating serotonin and platelet factor 4 (PF4) in the serum of patients with COVID-19 increase, which indicates degranulation of platelets [30].

Elevated levels of pro-inflammatory cytokines in COVID-19 may, inter alia, contribute to the increase in NOX activity [31], and consequently, the enhanced generation of superoxide radicals. As a result, local oxidative stress is created, which at the same time causes dysfunction of the vascular endothelium [32]. Studies by other authors have shown that the biomarkers of lung endothelial damage, angiopoietin-2 (Ang-2) and intercellular adhesion molecule-1 (ICAM-1) are significantly higher in deceased patients, which may be due to enhanced pulmonary vascular leakage, while pulmonary vascular endothelium increases the expression of ICAM-1, which can bind macrophages alveolar and intensify the production of inflammatory cytokines in the alveoli [33]. However, severe inflammation in COVID-19 patients is believed to be correlated with endothelial dysfunction, as seen in higher levels of thrombomodulin and sVCAM-1 (soluble vascular cell adhesion molecule-1), which is the only independent biomarker associated with mortality [34]. Another important mechanism contributing to the development of oxidative conditions in infection is the release of iron (III) ions by COVID-19 from hemoglobin, which may mediate the production of highly reactive hydroxyl radicals and increase the level of ferritin [35].

On the other hand, the results of this study indicate an equivocal antioxidant response of the organism, assessed at the systemic level in the blood plasma of patients with COVID-19. The activity of essential antioxidant enzymes, such as SOD-1 and SOD-2, which protect the body against overproduction and the action of the superoxide radical anions, is significantly reduced in the plasma of COVID-19 patients, most of all those who died as a result of the disease, but also those who survived it. At the same time, it is observed that the activity of both SOD isoenzymes is significantly lower in the plasma of patients who died as a result of the disease. On the other hand, immunohistochemical studies showed a higher level of the SOD-2 isoenzyme in the cells of vital organs, including the kidneys, and a lower level in the cells of the lungs [17], a critical organ in the course of COVID-19. Therefore, it can be suggested that as a result of the invasion of pathogens, the accumulation of enzymatic proteins in protein biosynthetic cells may occur.

In addition, the results of this study indicate opposite changes in the response of two basic cooperating antioxidant systems mainly responsible for the protection of lipids against oxidative modifications and for the maintenance of biologically significant sulfhydryl groups in the form of biologically active thiol groups of proteins. This counteracts the formation of oxidative stress in relation to GSH- and Trx-dependent systems [36], which confirms the dysregulation of antioxidant capacity in the body of patients with COVID-19. Reduced GSH levels are observed in the plasma of these patients. It is known that diminished GSH levels enhance cellular oxidation, which can exacerbate various disease states and immune dysfunctions leading to a greater susceptibility to viral infections, such as COVID-19 [37]. On the other hand, it is known that uncontrolled replication of the virus leads to oxidative damage in the lungs, which increases the viral load, thus exacerbating the infection [38]. Conversely, high GSH levels can prevent the virus from replicating efficiently, resulting in a lower viral load, and thus milder symptoms. A positive effect of GSH on the recovery of patients infected with COVID-19 was also observed, in agreement with previous findings that a high ROS/GSH ratio strongly correlates with the severity of disease symptoms and slower recovery [37]. Moreover, the role of glutathione in systemic reaction to COVID-19 infection was observed by other authors who suggested that GSH deficiency is the most likely explanation for the serious medical condition or death of COVID-19 patients [37]. It is known that the interaction of GSH with proteins, i.e., protein glutathionylation, is the main redox immune mechanism that affects the function of not only NF-kB, but also of proteins, such as signal transducer and activator of transcription 3 (STAT3), cAMP-dependent protein kinase (PKA), tumor necrosis factor receptor-associated factor 3 (TRAF3), and tumor necrosis factor receptor-associated factor 6 (TRAF6) [25]. Furthermore, the levels of thiol compounds that prevent or ameliorate damages to cellular components (e.g., proteins, lipids, and nucleic acids) are lower in COVID-19 patients than in healthy subjects and continue to decline as the patient’s condition worsens. Therefore, both the results of previous publications [39] and our study indicate that systemic oxidative stress associated with a disturbed thiol-disulfide balance play an important role in the pathogenesis of COVID-19.

Considering the above, the reduction in GSH levels, which inhibits the production of the most ROS-activated pro-inflammatory cytokines, supports the pro-inflammatory effects of Trx and promotes the increase in TNFα levels seen in COVID-19 patients. Moreover, it is known that GSH is necessary for the maintenance of an adequate level of interferon α (IFN-α) production by antigen presenting cells, and therefore for an efficient immune response against pathogens [40], which can inhibit viral replication. It was previously shown that the lower levels of IFN-α in the blood of seriously ill COVID-19 patients could be accompanied by elevated levels of pro-inflammatory cytokines (IL-5, IL-6, IL-10, and IL-13) [41]. Moreover, it has been found that abnormal antigen processing and diminished IL-12 secretion correlate with GSH deficiency in antigen-presenting cells, which promotes a type 1 T helper/type 2 T helper (Th1/Th2) to Th2 shift [42]. Furthermore, it has been shown that several genes important for antiviral activity, including the antiviral proteins, such as 2′-5′-oligoadenylate synthetase (OAS) [43] and myxovirus resistance 2 (MX2) [44], require the presence of GSH [45]. This supports suggestions that lowering GSH levels in COVID-19 patients may promote the development of infection, as with other viral diseases, while increasing GSH levels inhibited disease progression [46], and that viral infections generally induce the levels of glutathione and thioredoxin, as well as of the peroxiredoxin (PRDX) family [47]. In addition, despite the enhanced activity of enzymes cooperating with GSH, especially glutathione peroxidase, whose co-substrate is GSH, the reduced level of this tripeptide inhibits the effectiveness of its antioxidant activity. Moreover, it has been found that what unequivocally differentiate the group of surviving patients from those who died as a result of COVID-19 are only changes in the level of glutathione and the activity of GSHPx and GSSG-R, which indicates a significant influence of the glutathione-dependent system on the disease progression. Similar pattern of changes of the antioxidant parameters were also shown in another study [48].

Antioxidant effectiveness at the level of antioxidant enzymatic systems is supported by non-enzymatic antioxidants, which consist of cooperating ascorbic acid, tocopherols, retinol, and small components of the glutathione and thioredoxin systems (GSH and Trx) [49]. Since ascorbic acid, GSH and Trx, are not lipid soluble, acting only in a hydrophilic environment, they cannot protect lipids against ROS. In the plasma of patients with COVID-19, the levels of all the aforementioned antioxidants are reduced, except for Trx.

As a consequence, ascorbic acid cannot participate in the effective elimination of ROS; it also shows a reduced ability to regenerate oxidized forms of lipophilic antioxidants directly involved in the antioxidant protection of lipids. Therefore, under the oxidative conditions accompanying COVID-19, tocopherol and retinol exist predominantly in an oxidized form as evidenced by the lowered levels of their reduced forms in the plasma of patients with COVID-19 and cannot protect lipids from oxidation [50]. The consequence is enhanced lipid peroxidation in the plasma of patients, as shown by the level of the lipid oxidative fragmentation product, i.e., 4-HNE, but particularly the oxidative cyclization product, i.e., neuroprostanes. Another study indicated an increase in plasma lipid peroxidation (LPO) products level, but similar results were observed in recovered and deceased COVID-19 patients [51]. In our examinations, the observed elevated level of free 4-HNE was not statistically significant, probably due to the unique reactivity of this electrophilic aldehyde, which forms adducts with compounds containing nucleophilic structures, including GSH and proteins [52]. However, the observed lower levels of the free 4-HNE in the plasma of deceased patients compared to the levels in survivors is very interesting, as this could indicate enhanced binding of this bioactive aldehyde to the plasma proteins. Since the levels of 4-HNE protein adducts, notably of the aldehyde bound to histidine residues, were increased in both groups of COVID-19 patients, it is certain that systemic lipid peroxidation occurred due to the SARS-CoV-2 infection, but it is also likely that the metabolism of the protein adducts and the aldehyde itself was to some extent different in the survivors when compared to the patients who died several days later. This could be due to the systemic stress, and the age difference between these two groups, since it is known that 4-HNE forms protein adducts in the blood vessels, notably in aorta, in an age-dependent way, peaking at the age of 65–70 [41]. Due to the fact that the results are in agreement with the previously observed differential dynamic changes of the 4-HNE-protein adducts [21] and the immunohistochemical findings of 4-HNE in the vital organs of the deceased patients [17], the authors believe that further studies in this direction could be important for a better understanding not only of COVID-19 itself, but also of other severe sepsis-like diseases.

On the other hand, it should be mentioned that 4-HNE could have been bound to its usual scavenger GSH, but the levels of 4-HNE-GSH adducts could not be quantified in plasma. However, the formation of 4-HNE-GSH adducts may be one of the reasons for the decreased level of GSH in patients with COVID-19. At the same time, the presence of this type of reaction may explain the lack of elevated GSSG levels in those patients, which is usually associated with a reduction in GSH levels under oxidative stress. An increase in the level of glutathione disulfide is only observed in patients who died as a result of the disease. This situation is accompanied by a decrease in GSSG-R activity, which reduces the possibility of disulfide reduction to glutathione, the amount of which, however, is also elevated in those patients who do not survive. At the same time, however, the activity of glutathione peroxidase, the action of which protects the lipids against oxidative modifications, is significantly reduced in the plasma of those patients who died. As a result, the observed significant increase in the level of the assessed peroxidation products (4-HNE and neuroprostanes) confirms the definitely enhanced generalized oxidative stress in the bodies of patients who died as a result of the infection.

Furthermore, it has been shown that the level of 4-HNE protein adducts is almost twice as high in the plasma of COVID-19 patients than in healthy subjects. At the same time, the oxidative stress observed in COVID-19 patients promotes the interaction of ROS with proteins, which results in an elevated level of protein carbonyl groups with fragmentation of peptide protein chains, as confirmed by metabolic consequences caused in COVID-19 patients. The results of this study may indicate this type of situation. The immediately available clinical data linking COVID-19 with oxidative stress is limited. In contrast, the results of preclinical studies, including experimental animal models of severe acute respiratory failure, suggest that overproduction of ROS and impairment of the antioxidant system play a major role in the pathogenesis of a COVID-19 infection, as well as in the progression and severity of the disease [36,53].

The results of this study also indicate that components of the Trx system, including Trx levels and TrxR activity, are increased in the plasma of COVID-19 patients. One of the important functions of Trx is to support the transcriptional activity of the nuclear factor NFkB by promoting its interaction with DNA [37,54] and stimulating the biosynthesis of pro-inflammatory cytokines, including tumor necrosis factor alpha (TNFα), which in turn stimulates the biosynthesis of TrxR, which in turn, by reducing the oxidized form of Trx, leads to enhanced transcriptional activity of NFkB [44,55]. These relationships correspond to the situation observed in many pathological conditions, including chronic obstructive pulmonary disease (COPD), in which it was found that elevated expression of Trx and TrxR significantly disturbs redox homeostasis [56]. It is known that in response to oxidative stress, many cell types, including virus-infected cells, enhance Trx biosynthesis, which may affect the host’s immune response [56,57].

Since the current study revealed that some redox balance parameters (e.g., SOD-1/SOD-2) were significantly more disturbed in patients who died than in survivors, it is reasonable to consider comorbidities among severely ill patients, although there were no differences of comorbidities between the deceased patient and survivors in our study. On the other hand, literature data indicate that patients with the most severe forms of COVID-19 tended to be burdened with cardiometabolic diseases, in particular diabetes, which, apart from hypertension, poses the greatest risk, as people with diabetes are more susceptible to diseases, especially respiratory diseases. Moreover, diabetic patients are usually overweight, which exacerbates chronic inflammation, adversely affecting the glycemic profile, but that was not the case with our patients. Chronic hyperglycemia and chronic inflammation enhance the risk of developing and severity of COVID-19 and, consequently, enhancing the risk of death [58]. In addition, some proteins, such as inhibitor of nuclear factor kappa B kinase subunit beta (IKKβ), protein kinase C (PKC), and Kelch-like ECH-associated protein 1 (Keap1) that are damaged by redox modifications are responsible for the development of diabetes. Therefore, the therapeutic strategies that indicate the potential direction of diabetes treatment are based precisely on the post-translational modification of these proteins. In addition, an appropriate diet and physical activity can be helpful, thanks to which, as is known, the activity of these antioxidants as Mn-SOD (SOD-2) and GSHPx is increased [59].

In summary, it should be stated that in the case of an infection, oxidative stress plays a double role due to the fact that ROS, owing to their non-specific nature and unique reactivity, are harmful to the pathogen as well as to the host [8]. Overproduction of ROS promotes the degradation of pathogens, and thus protects the host organism against their effects. However, it is known that elevated levels of ROS may modify host metabolism, and consequently, lead to higher host mortality [9]. Therefore, the bidirectional nature of redox changes observed in the bodies of people with COVID-19, including survivors and those deceased, with respect to the thioredoxin-glutathione system, confirms the possibility of an atypical host response to this infection. As a consequence, patients with COVID-19 may recover or die, depending on the different metabolic response of the particular patient’s body. This partly corresponds to the effects of disease progression seen in COVID-19 non-survivors, whose levels of lipid peroxidation products were lower than in the other patients. Therefore, it can be suggested that a lower oxidative response corresponds to a lower inflammatory response, which is, however, an important consequence of the host immune response aimed at survival.

## 4. Materials and Methods

### 4.1. Samples Collection

The plasma samples were collected from a group of 66 COVID-19 survivors (21 female and 45 men), mean age 65 (53–72) and from 22 patients with COVID-19 or those who died (11 female and 11 men), mean age 76 (66–83) treated in the Clinical Hospital Dubrava in Zagreb, serving as the national COVID-19 center, thus providing medical care for patients even if suffering from the most aggressive COVID-19. Based on the opinion of experienced clinicians, according to the overall status of the COVID-19 patients and request for oxygen supply, fourteen patients received an oxygen supply of more than 8 L/min, eight were supported by the high-flow, while three required mechanical ventilation. The control group consisted of 34 healthy donors (22 female and 11 men, mean age 39 (28–55) (Table 1). Results of blood laboratory tests of healthy subjects and patients with COVID-19 are outlined in Table 2. This study was conducted after obtaining the ethical approval 2020-1012-13 of the Clinical Hospital Dubrava in Zagreb. Patients and healthy control subjects were denied any use of the antioxidants, including vitamins A, C, and E before collection of the blood samples, while vitamins were also not used as supplements during hospitalization of the patients. 

Blood samples were drawn at the bedside upon admission to the hospital by venipuncture and collected into ethylenediaminetetraacetic acid (EDTA) tubes and were centrifuged at 3000× *g* in 4 °C for 20 min to separate the plasma. The antioxidant—butylhydroxytoluene was added to plasma samples, which were stored at −80 °C until analysis.

### 4.2. Methods

#### 4.2.1. Antioxidant Parameters

##### Determination of Protein Antioxidants

The activity of glutathione peroxidase (GSH-Px) was determined with the use of the indirect method [60] based on the following two coupled reactions: Glutathione oxidation and glutathione reduction with NADPH oxidation to NADP. As a result, reduced light absorption was observed at a wavelength of 340 nm. The reaction was performed at 37 °C and pH = 7.6. The TRIS-HCl mixture was the control sample. The amount of enzyme that oxidizes 1 mmol of NADPH within 1 min was adopted as the unit of activity and expressed per ml of plasma.

The indirect method, which is based on the reduction of the oxidized form of GSH at the expense of the oxidation of NADPH to NADP, was used for the determination of the activity of glutathione reductase (GSSG-R) [61]. NADPH oxidation causes reduction in light absorption at a wavelength of 340 nm. The decrease in absorbance was measured over 1 min against a control containing phosphate buffer (0.02 M; pH = 7). The obtained results were presented in terms of 1 mL of plasma.

The level of thioredoxin (Trx) was measured using ELISA [62]. The preparation of the plasma sample started with an overnight incubation with a primary anti-thioredoxin antibody (Abcam, Cambridge, MA, USA). Then, a 1:100 dilution of goat anti-rabbit/mouse antibody (EnVision + Dual Link/HRP) (Agilent Technologies, Santa Clara, CA, USA) was added to the sample and incubated for 1 h. A solution of TMB (0.1 mg/mL) in a citric buffer with 0.012% hydrogen peroxide was used as a chromogen. Sulfuric acid (2 M) was used to stop the reaction. The samples were prepared on ELISA plates (Nunc Immuno Maxisorp, Thermo Scientific, Waltham, MA, USA). Spectral absorption was measured at 450 nm with the reference filter set to 620 nm and expressed as µg/mL.

The activity of thioredoxin reductase (TrxR-EC.1.8.1.9) was assessed colorimetrically at 412 nm with the use of a commercial test kit (Sigma-Aldrich, St. Louis, MO, USA) according to the instruction [63]. The Trx-R activity is expressed as U/mL of plasma. 

The spectrophotometric method (a wavelength of 480 nm) was used to determine the activity of superoxide dismutase—SOD-1 (Cu,Zn-SOD-EC.1.15.1.1) [64] and manganese-dependent superoxide dismutase—SOD-2 (Mn-SOD-EC.1.15.1.1) [65]. The measurement of activity was performed compared to the control group in the form of a carbonate buffer solution with pH = 10.2 (containing a 100 µM EDTA solution). The specific activity of Cu,Zn-SOD is presented as U/mL of plasma. 

##### Determination of Low Molecular Antioxidants

Capillary electrophoresis (CE) was used to measure reduced GSH [66] content in plasma. The procedure of sample preparation consisted of protein precipitation with ACN and centrifugation. Immediately, the supernatant was measured using CE. An ultraviolet detector set at 200 ± 10 nm was used for the performance of separations. The level of reduced GSH is expressed as nmol/mL of plasma.

The levels of fat-soluble (A and E) and water-soluble vitamins (C) were determined with the modified method [67,68] using HPLC (Agilent Technologies, Santa Clara, CA, USA) with a DAD detector. The wavelengths for vitamins A, E, and C are 325, 294, and 250 nm, respectively. The process of sample preparation for the determination of vitamins A and E included the steps of extraction with hexane, evaporation, and dissolution in ethanol prior to the performance direct injection on the column. To determine the level of vitamin C, metaphosphoric acid (1:1) was added to the sample, leading to the precipitation of protein. After centrifugation, the supernatant was injected onto the column. The vitamin levels were estimated using calibration curves with ranges of 0.5–25 μg/mL (r^2^ = 0.9985), 10–500 ng/mL (r^2^ = 0.9989), and 0.5–20 μmol/mL (r^2^ = 0.9987) for vitamins A, E, and C, respectively.

#### 4.2.2. Lipid Peroxidation Products Determination

The low molecular aldehyde, i.e., 4-HNE, generated during phospholipid fragmentation was determined as an *O*-pentafluorobenzyl-oxime (*O*-PFB-oxime) derivative using the GC-MS method based on Tsikas et al. [69] with minor modifications. A derivatizing agent, i.e., *O*-(2,3,4,5,6-pentafluoro-benzyl) hydroxylamine hydrochloride (0.05 M in PIPES buffer), was added to the samples, with d3-4-HNE as the internal standard. The samples were incubated for 24 h in room temperature. This was followed by deprotonation of aldehydes by methanol and extraction with hexane. Thereafter, a hexane layer that contained *O*-PFB-oxime aldehyde derivatives was removed, transferred into borosilicate tubes, and evaporated under a stream of argon. Then, *N*,*O*-bis(trimethylsilyl)trifluoroacetamide in 1% trimethylchlorosilane was added and incubated for 15 min in a temperature of 80 °C. Aldehydes were separated on an HP-5 ms capillary column with a temperature gradient and analyzed using a GC-MS system (7890A GC-7000 with triple quadrupole mass spectrometer, Agilent Technologies, Palo Alto, CA, USA). The following ions were used: *m*/*z* 242.0 and 204.0 for 4-HNE-PFB-TMS and IS derivatives, respectively. The linear dynamic ranges were 0.05–20.00 nmol/mL.

Neuroprostanes were assayed using the modified LC-MS methods proposed by Coolen and Dupuy [70,71]. The process of sample preparation for neuroprostanes determination consisted of the following main steps: Hydrolysis and sample purification with the use of SPE columns (SEP-PAK C18; Waters Corporation, Milford, MA, USA). The prepared samples were injected onto a column that is part of Agilent 1290 UPLC system equipped with a triple quadrupole mass spectrometer with an electrospray source (ESI). The samples were analyzed in the negative-ion mode using the following MRM transitions: *m*/*z* 377.0→153.0 and 357.2→197.1 for neuroprostanes and 8-isoPGF2 α-d4, respectively. The linear dynamic range was 60–6000 pg/mL.

#### 4.2.3. Determination of Protein Oxidative Modifications

The enzyme-linked immunosorbent assay (ELISA) is a method commonly used for the detection of 4-HNE bound to proteins. It is considered as the most probable form of 4-HNE in living systems. The levels of 4-HNE-protein adducts in plasma [72] were measured using the anti-4-HNE-protein monoclonal mouse antibody (Invitrogen, Burlington, Canada) and the goat anti-mouse EnVision+ Dual Link/HRP solution (1:100) (Agilent Technologies, Santa Clara, CA, USA) as secondary antibodies. The levels of 4-HNE-protein adducts were assessed on the basis of a calibration curve (0.5–25 pmol/mg BSE; r2—0.9983) and converted into mg of protein.

The protein oxidative modifications (carbonyl groups—CBO) were determined with the use of the spectrophotometric method at a wavelength of 370 nm. Then, 2,4-dinitrophenylhydrazine was used for the preparation of the sample and the content was expressed as nanomoles per mg of protein [73].

### 4.3. Statistical Analysis

The data obtained in this study were expressed as mean ± SD. For comparisons between groups, one-way analysis of variance (ANOVA) was used, followed by a post hoc Tukey test. The value of *p* < 0.05 was considered as statistically significant. The Mann-Whitney U test and the Wilcoxon signed-rank test were used for the comparison of laboratory tests. All the statistical analyses were performed using Statistica software (Statistica 13.3, StatSoft, Cracow, Poland). The post-hoc power of our dichotomous study was 100% (https://clincalc.com/stats/Power.aspx (accessed on 21 March 2022)).

To evaluate clustering trends and separation of the three analyzed data sets of control, COVID-19 recovered patients, and COVID-19 deceased patients, we performed principal component analysis (PCA) and partial least squares-discriminate analysis (PLS-DA) using Metaboanalyst, version 5.0 online tool (https://www.metaboanalyst.ca/ (accessed on 8 August 2022)) [74]. The most relevant parameters responsible for driving the separation of examined groups were selected according to variable importance in projection (VIP) (Supplementary Materials, Figures S1–S3).

## 5. Conclusions

Due to the reduced antioxidant capacity in the plasma of COVID-19 patients found in this study, particularly with regard to GSH and related low-molecular-weight antioxidants, especially lipophilic, it should be concluded that the organisms of patients with the severe form of COVID-19 were not able to counteract oxidative modifications. This is clearly confirmed by the results of the PLS-DA model confirming a significant role in the development and consequences of COVID-19 of the deficiency of GSH-related antioxidants and the consistently increased generation of lipid peroxidation products. Moreover, metabolic comparison of surviving and deceased patients indicates a likely fatal outcome that can be attributed to the inability to withstand oxidative changes, especially lipid peroxidation. As a consequence, there is an accumulation of electrophilic molecules that transfer the effects of persistent oxidative stress to other bioactive molecules, particularly proteins important for the proper functioning of cells and the systemic response to SARS-CoV-2 infection, including the response to the observed inflammation. Therefore, to reduce these negative effects of infection, the administration of antioxidants, particularly lipophilic agents, could be suggested to assist the body in counteracting the sepsis-like effects of aggressive COVID-19. Our results suggest that preventive and/or supportive use of antioxidants, may affect treatment outcomes, particularly in severely ill patients requiring high doses of oxygen or mechanical ventilation.

## Figures and Tables

**Table 1:** Demographic and clinical characteristics of patients with COVID-19 compared to healthy subjects.

	Healthy Control	COVID-19 Recovered	COVID-19 Deceased
Age (years)	38.5 ± 9.3	65.4 ± 8.1 ^a^	75.7 ± 7.6 ^a,b^
Sex	22F 11M	21F 45M	11F 11M
Body Mass Index	26.8 ± 4.4	28.5 ± 3.4	27.1 ± 6.8

^a^—significant to the healthy control group; ^b^—significant to the group of recovered patients.

**Table 2:** Comparison of some laboratory data of patients with COVID-19 in respect to normal values.

	Normal Range	COVID-19 Recovered	COVID-19 Deceased
WBC [10^3^/μL] Neutrophils [%] Platelets [10^3^/μL]	4.00–10.00 40.0–72.0 150–400	10.15 ± 3.27 80.67 ± 7.71 255.97 ± 93.21	10.86 ± 3.64 84.82 ± 5.87 * 199.35 ± 52.78 *
Blood oxygen saturation [%]	>95%	92.49 ± 3.47	87.13 ± 8.43 *
Ferritin [μg/L]	11–336	959 ± 573	1007 ± 486
PCT [ng/mL]	<0.1	0.58 ± 0.7	1.71 ± 1.87
LDH [U/L]	140–280	355 ± 147	456 ± 224
CRP [mg/L]	0.00–5.00	114.51 ± 61.88	172.06 ± 71.23 *
IL-6 [pg/mL]	0–43.5	125 ± 107	266 ± 179

*—significant (*p* < 0.05) difference to the values of recovered patients.

## Data Availability

The data presented in this study are contained within the article.

## Supplementary Materials

The following supporting information can be downloaded at: https://www.mdpi.com/article/10.3390/molecules27165323/s1. Figure S1: Two-dimensional principal component analysis (2D PCA) scores plot of all parameters examined in the study and determined in the plasma of healthy subjects (*n* = 34) [Control], recovered COVID-19 patients (*n* = 66) [COVID-19 R], and deceased COVID-19 patients (*n* = 22) [COVID-19 D]; Figure S2: Partial least squares-discriminate analysis (PLS-DA) plot of all parameters examined in the study and determined in the plasma of healthy subjects (*n* = 34) [Control], recovered COVID-19 patients (*n* = 66) [COVID-19 R], and deceased COVID-19 patients (*n* = 22) [COVID-19 D]; Figure S3: Graphical presentation of variable importance in projection (VIP) of all parameters examined in the study and determined in the plasma of healthy subjects (*n* = 34) [Control], recovered COVID-19 patients (*n* = 66) [COVID-19 R], and deceased COVID-19 patients (*n* = 22) [COVID-19 D].

## Sample Availability

Samples of the compound arenot available from the authors.
