# Supplementary material for: The Impact of Severe COVID-19 on Plasma Antioxidants
Source: Molecules. 2022 Aug 21;27(16):5323. doi: 10.3390/molecules27165323 (PMC9416063; doi:10.3390/molecules27165323)
Supplement: Supplementary file 1 [file molecules-27-05323-s001.zip › molecules-1848176-supplementary.pdf]

Supplementary Materials

The Impact of Severe COVID-19 on Plasma Antioxidants

Neven Žarković [1,*], Anna Jastrząb [2], Iwona Jarocka-Karpowicz [2], Biserka Orehovec [3], Bruno Baršić [3], Marko Tarle [3], Marta Kmet [3], Ivica Lukšić [3,4], Wojciech Łuczaj [2] and Elżbieta Skrzydlewska [2]

[1] Laboratory for Oxidative Stress (LabOS), Ruđer Bošković Institute, HR-10000 Zagreb, Croatia
[2] Department of Analytical Chemistry, Medical University of Bialystok, A. Mickiewicza 2D, 15-222 Bialystok, Poland
[3] Clinical Hospital Dubrava, 10000 HR-Zagreb, Croatia
[4] University of Zagreb School of Medicine, HR-10000 Zagreb, Croatia
* Correspondence: zarkovic@irb.hr

Principal component analysis (PCA) was carried out using the complete data set of all examined parameters with the normalized and auto-scaled values of their level/activities. In the PCA principal components (PC1 and PC2) represent the variation in the variables. It is highly probable that PC1 accounted for 17.6% is associated with a diseased state, and is responsible for observed discrimination of COVID-19 patients from healthy subjects (Figure S1).

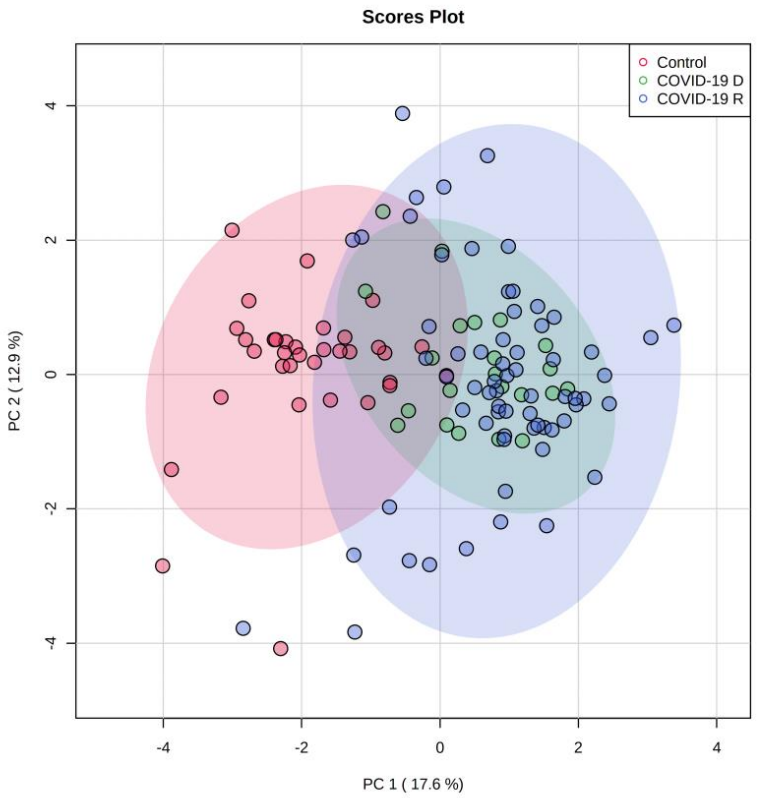

Figure S1. Two-dimensional principal component analysis (2D PCA) scores plot of all parameters examined in the study and determined in the plasma of healthy

subjects (n = 34) [Control], recovered COVID-19 patients (n = 66) [COVID-19 R], and deceased COVID-19 patients (n = 22) [COVID-19 D].

Subsequently, a partial least squares-discriminate analysis (PLS-DA) was used as statistical method to establish a discrimination model. Obtained PLS-DA model of the normalized data sets allows differentiation of the two groups of COVID-19 patients and healthy controls (Figure S2).

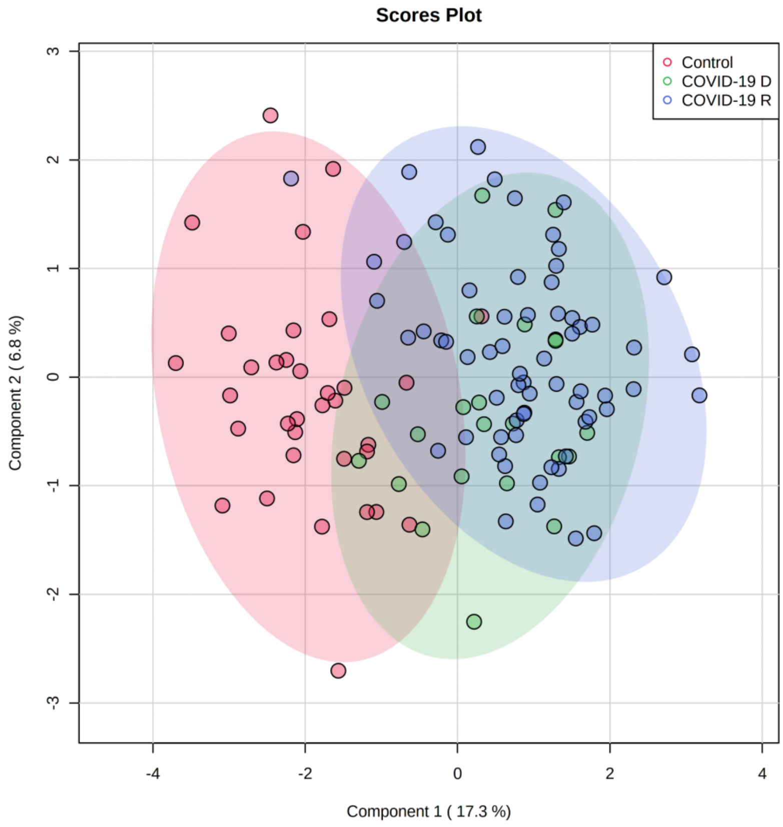

Figure S2. Partial least squares-discriminate analysis (PLS-DA) plot of all parameters examined in the study and determined in the plasma of healthy subjects (n = 34) [Control], recovered COVID-19 patients (n = 66) [COVID-19 R], and deceased COVID-19 patients (n = 22) [COVID-19 D].

In the PLS-DA model, the variable importance of the factors in the projection (VIP) coefficient reflects the contribution of each parameter in the observed discrimination of groups. Variables with VIP score close to or greater than one are regarded as significant and therefore considered for quantitative analysis of variation. Using this criterion, six parameters analyzed in this study were identified, which were driving the separation of examined groups. These parameters include vitamin E, GSSG-R, 10-F4t-NeuroP, GSH, vitamin A, GSH-Px and adducts of 4-HNE-proteins (Figure S3).

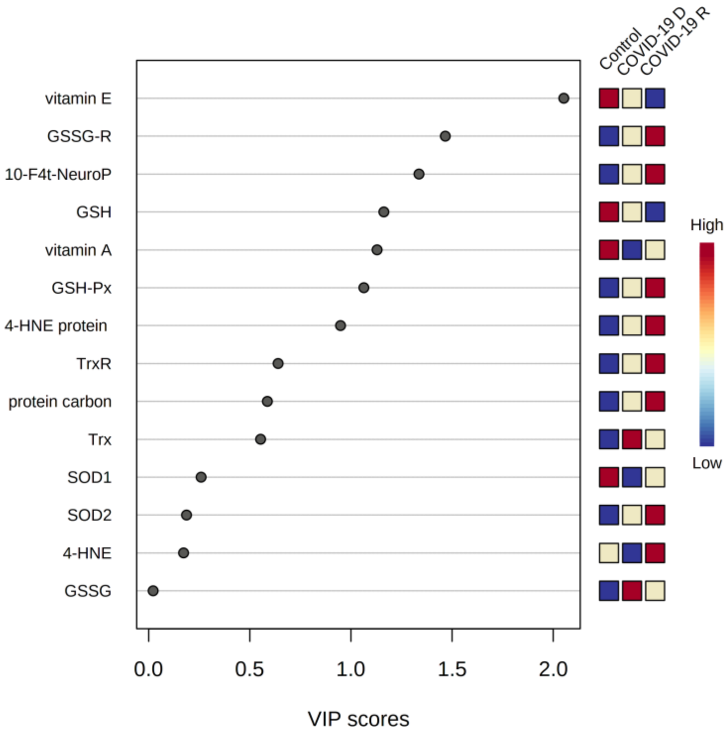

Figure S3. Graphical presentation of variable importance in projection (VIP) of all parameters examined in the study and determined in the plasma of healthy subjects (n = 34) [Control], recovered COVID-19 patients (n = 66) [COVID-19 R], and deceased COVID-19 patients (n = 22) [COVID-19 D].
